# Insights into the Gene Expression Profile of Classical Hodgkin Lymphoma: A Study towards Discovery of Novel Therapeutic Targets

**DOI:** 10.3390/molecules29153476

**Published:** 2024-07-25

**Authors:** Abdulaziz A. Aloliqi

**Affiliations:** Department of Basic Health Sciences, College of Applied Medical Sciences, Qassim University, Buraydah 52571, Saudi Arabia; aaalieky@qu.edu.sa

**Keywords:** classical Hodgkin lymphoma, microarray data analysis, docking, simulations, gene expression, drug target, cancer therapy

## Abstract

Classical Hodgkin lymphoma (cHL) is a common B-cell cancer and a significant health concern, especially in Western and Asian countries. Despite the effectiveness of chemotherapy, many relapse cases are being reported, highlighting the need for improved treatments. This study aimed to address this issue by discovering biomarkers through the analysis of gene expression data specific to cHL. Additionally, potential anticancer inhibitors were explored to target the discovered biomarkers. This study proceeded by retrieving microarray gene expression data from cHL patients, which was then analyzed to identify significant differentially expressed genes (DEGs). Functional and network annotation of the upregulated genes revealed the active involvement of matrix metallopeptidase 12 (MMP12) and C-C motif metallopeptidase ligand 22 (CCL22) genes in the progression of cHL. Additionally, the mentioned genes were found to be actively involved in cancer-related pathways, i.e., oxidative phosphorylation, complement pathway, myc_targets_v1 pathway, TNFA signaling via NFKB, etc., and showed strong associations with other genes known to promote cancer progression. MMP12, topping the list with a logFC value of +6.6378, was selected for inhibition using docking and simulation strategies. The known anticancer compounds were docked into the active site of the MMP12 molecular structure, revealing significant binding scores of −7.7 kcal/mol and −7.6 kcal/mol for BDC_24037121 and BDC_27854277, respectively. Simulation studies of the docked complexes further supported the effective binding of the ligands, yielding MMGBSA and MMPBSA scores of −78.08 kcal/mol and −82.05 kcal/mol for MMP12-BDC_24037121 and −48.79 kcal/mol and −49.67 kcal/mol for MMP12-BDC_27854277, respectively. Our findings highlight the active role of MMP12 in the progression of cHL, with known compounds effectively inhibiting its function and potentially halting the advancement of cHL. Further exploration of downregulated genes is warranted, as associated genes may play a role in cHL. Additionally, CCL22 should be considered for further investigation due to its significant role in the progression of cHL.

## 1. Introduction

In 1832, Thomas Hodgkin identified classical Hodgkin lymphoma (cHL), a cancer of the lymphatic system that affects normal B-cells [1]. It occurs at a rate of 2–3 instances per 100,000 individuals annually in prosperous countries. Approximately 25–40% of cases of cHL may be causally linked to Epstein–Barr virus infections. These cases may also include individuals with a personal history of autoimmune diseases or immunological deficiencies, including HIV infection and cancers that develop from low-grade B-cell lymphoma, mostly chronic lymphocytic leukemia [2]. Globally, cHL is responsible for around 30% of lymphoid malignancies and 1% of all cancers [3]. Studies on the epidemiology of cHL show that the occurrence varies significantly depending on factors such as age, sex, ethnicity, region, and socioeconomic level. Caucasians had the highest occurrence, followed by African Americans and Hispanics; Orientals had the lowest prevalence [4]. The International Agency for Research on Cancer’s data from 2008 indicates that the incidence of the disease in East Asia and Western Europe was 0.4 and 2.3 per 100,000 people annually [2]. Young individuals are typically affected by cHL, which exhibits a bimodal age distribution, peaking in two age groups: early adulthood (ages 15 to 35) and late adulthood (ages 55 and above). While the difference narrows in older individuals, there is a modest male predominance in most age groups [5].

Over the last several decades, overall death rates for cHL have declined due to advancements in treatment modalities such as stem cell transplantation, radiation therapy, and chemotherapy [6]. Despite these improvements, cHL still accounts for a significant portion of lymphoma-related fatalities worldwide. In affluent nations, five-year survival rates for cHL typically range from 80% to 90%. Variables such as age, stage of diagnosis, presence of B cell symptoms (fever, night sweats, weight loss), and response to treatment all influence the survival rate [2]. cHL is highly curable, and innovative treatment approaches have led to high cure rates, resulting in an improved prognosis over the decades [1]. Estimates of 5-year overall survival (OS) for favorable and unfavorable risk early-stage disease, using the European Organisation for Research and Treatment of Cancer staging criteria, range from 99.4% to 96.0% [6]. This difference, though minor, is statistically significant (*p* < 0.001). The 5-year OS for advanced-stage illness varies from 56% to 89%. Despite these advancements, important clinical needs remain unmet due to the unexplained pathophysiology of cHL [6]. New treatment approaches are required to prevent or cure relapsed/refractory (R/R) disease, reduce treatment-related morbidity, improve quality of life (QoL), and enhance patient outcomes, especially for individuals over 60 [6].

The emergence of bioinformatics has provided answers to many queries and has played a pivotal role in identifying key genes related to complex diseases [7,8,9]. Numerous research studies have utilized bioinformatics analysis to investigate various complex diseases and identify therapeutic and diagnostic markers associated with complex abnormalities [9,10]. Multiple research studies aim to identify key genes specific to cHL that can serve as therapeutic targets and diagnostic markers. These studies focus on understanding the genetic underpinnings of cHL to develop more effective treatments and improve patient outcomes [10,11,12].

In this research study, a similar strategy was used. A microarray dataset consisting of gene expression profiles of cHL patients and healthy individuals was analyzed, and differentially expressed genes (DEGs) were identified between the two groups. The genes that were highly expressed in cHL patients compared to healthy individuals were further analyzed using various downstream analyses, including gene set enrichment analysis (GSEA) and network analysis [13,14,15]. These analyses explored the role of these genes in cHL patients and their correlation with cHL progression. Furthermore, the output of these analyses revealed some key genes associated with cHL progression, which were further validated using reference experimental data from the Cancer Genome Atlas and Genotype Tissue Expression (GTEx) Project databases [16,17]. The study further explored the potential of using the identified key gene as a therapeutic target by virtually blocking its function with a variety of anticancer compounds introduced into its active site. This process resulted in the identification of several potential compounds that can be considered the best inhibitory candidates for the key gene. 

This research has two main findings: the discovery of a novel therapeutic target related to cHL and the retrieval of key compounds that can be used as therapeutic inhibitors against the cHL progressor gene. These findings contribute significantly to the understanding of cHL and present new avenues for the development of targeted therapies to improve patient outcomes. 

## 2. Research Workflow

**Figure 1:**
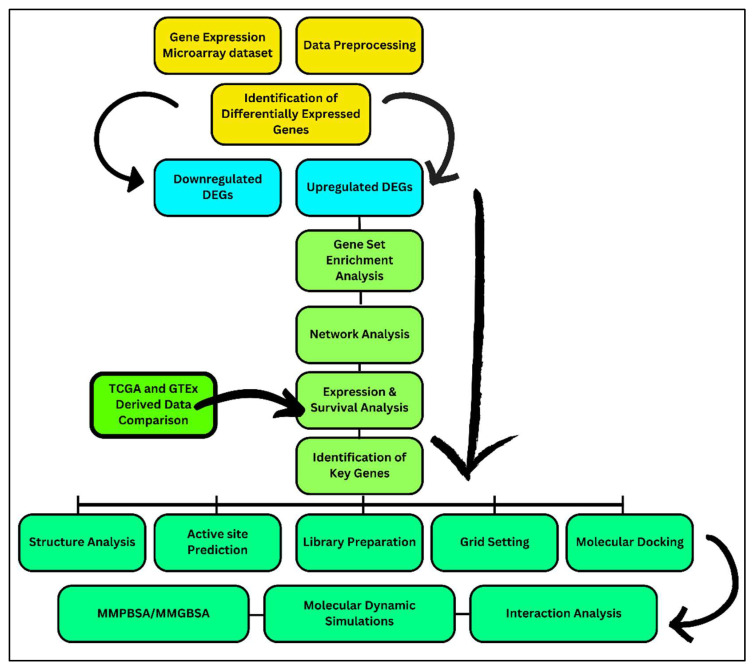
Research workflow. This study commenced with the retrieval of gene expression microarray datasets related to cHL and healthy individuals. The data were preprocessed using various statistical tools to obtain the most significant gene expression values. Differentially expressed genes were identified, revealing sets of upregulated and downregulated genes in cHL. Further analysis of the upregulated genes was conducted with respect to their involvement in different pathways using GSEA analysis. Additionally, network analysis was performed on the upregulated genes to identify deep connections among them. Significant interactions were found between the top two upregulated genes, highlighting their effective role in cHL. This hypothesis was validated by further exploring the roles of key genes using reference databases. The analysis identified key genes with significant roles in the progression of cHL. Subsequently, structural analyses were performed, where the three-dimensional protein structure of the target gene was retrieved, minimized, and evaluated for stability. The active site was predicted, and its coordinates were calculated for the grid box setting. Ligands were retrieved and minimized for the docking protocol. Both the ligands and the protein were selected for docking, and the binding potentials were calculated, followed by interaction analysis between the docked entities. Finally, simulations were conducted on the docked complexes to evaluate their behavior, respectively.

The overall work flow of research includes the identification of DEGs from microarray datasets specific to classical Hodgkin lymphoma (cHL), followed by the enrichment of DEGs using various approaches and finding the most responsible gene associated with lymphoma. Furthermore, docking of the concerned gene with inhibitor compounds was performed and proceeded with molecular dynamic simulations. The overall workflow is shown in Figure 1.

### 2.1. Microarray Dataset Selection Criteria

Microarray data were gathered from GEO (https://www.ncbi.nlm.nih.gov/geo/ accessed on 2 February 2024) using search terms such as “Hodgkin lymphoma.” The dataset had to originate from *Homo sapiens* ancestry to meet the selection criteria. Additionally, all samples in the datasets were obtained directly from patients’ tonsils and blood; cell line-based experiments were not included. Moreover, the chosen dataset lacked any gene knockdown, induced gene expression, mutations, therapy, or medication. To generate statistically meaningful results, all datasets were chosen with an adequate number of control and cHL samples. A dataset with the accession ID (GSE12453) was chosen for microarray analysis based on the aforementioned selection criteria. Table 1 contains the dataset’s details, including the platform, samples, and accession number. The dataset included 67 samples with various abnormalities, of which 10 samples linked to healthy individuals and 12 samples pertaining to cHL patients were selected for examination based on relevant research. The main objective of our study was to identify the key biomarkers associated with cHL and propose a drug discovery strategy to oppose its function and stop the progression of cHL.

### 2.2. Microarray Data Analysis

Microarray data analysis was solely performed using R-Studio v4.4.1. Different Bioconductor packages were used to generate the desired outputs. The raw expression data generated from microarray technology was preprocessed to obtain the most significant DEGs. Expression data were visualized before and after preprocessing to obtain a clear idea of the best preprocessing techniques. Log2 transformation was performed to reduce the variance of the raw data, followed by Robust Multi-Array Average (RMA) normalization using the Biobase Bioconductor R package [18]. Different data visualization R packages, such as ggplot2 and pheatmap, were used to better understand the structure and quality of the data and to compare the raw and normalized data effectively. The HG-U133_Plus_2 R-package was used to annotate the probe IDs with gene symbols and names [19]. Furthermore, low-intensity probes were filtered, and duplicates were eliminated to prevent noise in the data due to nonspecific hybridization of the probes. A linear regression model and eBayes were employed for the statistical analysis. To determine the DEGs in cHL samples relative to normal, a linear model was fitted using the limma R package [20]. Next, t-statistics, which produce the statistical significance value (*p*-value) for every DE gene, were computed by fitting the “Empirical Bayes” model using the eBayes function in R. Log2FC (Log2 fold change) thresholds (Log2FC < −1 and Log2FC > 1) and the adjusted *p*-value criterion of 0.05 were applied to obtain significant DEGs [21,22,23]. Enhanced volcano plots were used to visualize DEGs, with −Log10 (*p*-value) on the *y*-axis and Log2FC on the *x*-axis [24]. 

### 2.3. Gene Set Enrichment Analysis (GSEA)

Gene set enrichment analysis (GSEA) is a method that identifies whether predefined sets of genes show significant differences in expression between different biological conditions, aiding in understanding how biological pathways contribute to observed gene expression changes [13]. The obtained DEGs were subjected to gene set enrichment analysis (GSEA) to observe their involvement in different biological pathways. This analysis provides insight into which types of DEGs, whether upregulated or downregulated, are associated with specific pathways. This information helps us understand which types of DEGs are more closely related to pathways involved in lymphomas, offering valuable insights into the underlying biology of these diseases. The analysis was conducted using R-studio, where specific R packages such as “msigdbr” and “clusterprofiler” were utilized for smooth analysis [25,26]. Hallmark gene sets were specified from the Molecular Signature Database (msigdbr) to annotate our input genes. Furthermore, the Benjamini–Hochberg correction method was implemented, where the adjusted *p*-value was set to 0.01 just to obtain the most significant involved pathways [27]. DEGs, along with their specific logFC values, were provided as input files (ranked list), and the analysis was effectively performed. The results were visualized via bar plots and graphs using the ggplot2 R package [28].

### 2.4. Network Enrichment Analysis

Observing the GSEA results, it was found that the upregulated genes were mostly involved in pathways specific to lymphoma conditions. Network analysis was exclusively performed for the upregulated genes to examine the actual interaction between the genes. The STRING tool was utilized, with all the upregulated genes (logFC ≥ 1) provided as input. The STRING tool is an open web tool that integrates information from top biological databases [14]. It utilizes experimental and predicted data to identify the best associations between query genes. A statistical threshold of 0.7 was applied to the query data to identify the most promising associations. The resulting network was imported into Cytoscape software v 3.10.2 for further analysis according to the study design [15]. The Network Analysis module in Cytoscape was utilized to assess the interaction patterns and statistical data of elevated genes. Based on the results obtained from microarray data analysis, the top 5 upregulated genes with the highest logFC values were selected from the network and analyzed for interaction analysis. This was conducted to investigate which other upregulated genes are connected to them. The obtained associations were observed, and interactions of the top 2 upregulated genes were exported back to the STRING tool to check their significant correlations with their interacting nodes using a 0.7 confidence level. This pipeline will help identify the most significant interacting genes with our query genes (top upregulated genes) and lead to the identification of key genes involved in lymphoma.

### 2.5. TCGA and GTEx Derived Data Comparison

As mentioned, network analysis helps us identify the key genes involved in cHL and also provides information about the dependence of other genes on the top highly regulated genes. The significant genes obtained from network analysis were cross-validated for their functions in other carcinomas using the GEPIA 2 tool [29]. GEPIA 2 is an online bioinformatics tool that incorporates already-published expression data for different malignancies from The Cancer Genome Atlas (TCGA) and Genotype Tissue Expression (GTEx) databases [16,17]. This approach will provide us with an accurate insight into the selected gene to be considered a target gene causing cHL progression. 

### 2.6. Survival Analysis and Identification of Target Gene

This analysis is mostly conducted using the GEPIA 2 tool. As mentioned, a vast amount of expression data from various malignancies is integrated into this tool, and performing survival analysis on our query gene can help us understand the effect of our selected gene on patient health. In survival analysis, the query gene is used as input, and based on existing data, comparisons are made between normal and case individuals. This analysis provides insights into the effect of a specific gene on individuals’ overall survival when it is highly expressed or expressed at lower levels [30].

### 2.7. Structure Analysis of Target Protein and Active Site Prediction

Confirming the target gene as a progression factor in cHL from the above analysis, it was necessary to search for specific inhibitors and effectively dock them into the active site of the protein. This action would halt its function, thereby suppressing the progression of cHL. To achieve this goal, several mandatory steps should be considered, as discussed further. The three-dimensional structure of the target protein was obtained from the UniProt database and was subjected to structure refinement using the Galaxy Refine tool [31,32]. GalaxyRefine employs a comprehensive approach to protein structure refinement, combining energy minimization, ensemble optimization, solvent exposure considerations, homology-based modeling, and structure validation techniques to produce high-quality, stable, and biologically relevant protein structures. The refined structure is essential for subsequent docking studies because it relaxes the protein by reducing internal steric clashes and hindrances between amino acids. Furthermore, a secondary structure assessment of protein structure was performed using the psipred tool. Stability analysis was performed using the PDBsum tool and ERRAT, which is crucial for ensuring the protein’s stability before docking [33,34]. Unstable proteins are more likely to unfold or change shape, which can hinder their ability to bind to their intended ligands. Active site prediction of the protein is also an important step in computational drug discovery. This prediction was conducted using an online tool called CASTp, which identifies hollow areas in the protein structure and identifies the amino acids associated with the active site using Voronoi decomposition, followed by Delaunay triangulation, also known as the alpha-based approach [35]. This prediction helps to accurately position the grid box when performing docking between the ligand and the receptor protein. By following these steps, optimized docking and acceptable results can be achieved in computational drug discovery approaches.

### 2.8. Ligands Selection

Choosing specific ligands related to the mentioned query protein is an essential step to ensuring research integrity. The target protein obtained from the above analyses was confirmed as a crucial biomarker of cHL. Therefore, ligands associated with anticancer activities were selected for the docking protocol. The Asinex database was utilized to retrieve ligands shortlisted by the Targeted Oncology Library (TOL) [36]. Asinex specializes in providing chemical compound libraries and related services to pharmaceutical, biotechnology, and academic research institutions. They offer a variety of compound libraries tailored for different purposes, including drug discovery and development. The TOL is a specialized collection of chemical compounds designed specifically for oncology research. These libraries typically consist of small molecules selected or designed based on their potential to target specific molecular pathways or mechanisms relevant to cancer. The library was retrieved, and all compounds were energy-minimized using the PyRx tool to optimize them for docking studies.

### 2.9. Molecular Docking

To conduct docking studies, the ligands were converted into pdbqt format. The target protein was prepared by removing water molecules and adding polar hydrogens using UCSF Chimera [37]. The structural energy was minimized using 1000 steps of steepest descent and conjugate gradient methods, with a step size of 0.02 Å. The side chains and backbone of the protein were parameterized using the AMBER f14SB38 force field [38]. Grid box coordinates for the protein were determined based on the location of amino acids contributing to the active site using AutoDockTools 1.5.7 [39]. The prepared target protein was then converted into pdbqt format, and docking was performed using the PyRx tool [40]. The docking results and the interactions between the ligands and the target protein were visualized using Discovery Studio [41].

### 2.10. Molecular Dynamic Simulations

For molecular dynamics (MD) simulations, the best-docked complexes were chosen. We ran simulations using the Amber12 program [42]. The AMBER FF99SB force field was employed to model the target protein, while the RESP fitting procedure was used to obtain the inhibitor charges [43]. Using the Antechamber module in AmberTools12, topology preparation files for ligands were constructed with the general AMBER force field (GAFF) [44]. The complexes were then neutralized with 10 Na^+^ ions and solvated in a truncated octahedral box of TIP3P water molecules with a buffer size of 12 Å [45]. Default protonation states for protein residues were applied using Amber12 [46]. The SANDER module of the Amber12 package was utilized to perform the MD simulations and energy minimization for each system. Initially, 10,000 cycles of minimization (MAXCYC) were carried out on all atoms in the system, combining the first 700 steps of steepest descent (NCYC) with 9300 steps of conjugate gradients to relieve negative steric interactions and approach an energy minimum. After energy minimization, position restraints were applied at constant volume (NVT) for 100 ps with a force constant of 10 Kcal/mol at 100 K temperature, followed by constant pressure (NPT) for 100 ps with a force constant of 1 Kcal/mol at 300 K temperature. 

The next phase involved an equilibration at 300 K and constant pressure (NPT). This step eliminated the constraint force and had a duration time of 100 ps and a time step of 2 fs. By using this method, the water can reach an equilibrium density and equilibrate around the protein. Temperature was regulated using Langevin dynamics during the position constraints and equilibration phases. Ultimately, a production run lasting 100 ns was executed at constant pressure (NPT ensemble) using a 2 fs time step and isotropic position scaling (ntp = 1) at 300 K. The temperature was managed using the Berendsen thermostat (ntt = 1). The SHAKE algorithm was used to constrain all bonds involving hydrogen atoms (ntc = 2, ntf = 2). Periodic boundary conditions were applied throughout (ntb = 1). Long-range electrostatic interactions were handled using the particle mesh Ewald method, with a nonbonded cut-off of 10 Å [47]. The tleap module of Amber12 was used to coordinate calcium and zinc ions to relevant residues. LigPlus was utilized to create images of the ligands within the protein’s active site [48].

### 2.11. MM-PB/GBSA Calculations

The molecular mechanics Poisson–Boltzmann/surface area (MM-PBSA) method is considered an attractive approach for drug design since it can be applied to biological molecules like proteins and DNA, as well as small and medium-sized chemical compounds. Despite the time-consuming nature of thermodynamic integration (TI) and free energy perturbation (FEP), MM-PBSA offers a rapid and practical molecular dynamics-based approach for estimating binding free energy. Additionally, MM-PBSA does not rely on empirical parameters in its free energy computations, unlike the linear interaction energy (LIE) method. The MM-PBSA method is a potential strategy for ranking highly dissimilar compounds from database searches for binding to a specific site because it does not require a training set to establish empirical parameters, unlike the LIE method. Therefore, one can reliably simulate a protein–DNA complex by combining this approach with molecular docking and molecular dynamics simulations. In this investigation, the MM-PBSA approach was used to calculate binding energies using Amber12 [49]. For binding free energy calculations, 400 snapshots were taken from the MD simulation trajectories. The counter-ions and water molecules were removed before analysis [49]. 

In summary, the binding free energy is:

∆G_binding_ = G_complex_ − [G_protein_ + G_ligand_] (1)

Each free energy term in Equation (1) is computed using Equation (2):

*G* = E_gas_ + G_solvation_ − T∆S (2)

T∆S denotes the entropy term. The term E_gas_ refers to the vacuum molecular mechanical energy in the gas phase, which is further subdivided into internal, van der Waals, and electrostatic energy components. The solvation-free energy, ∆G_solvation_, is calculated using continuum solvent techniques. As indicated by Equation (3), ∆G_solvation_ can be divided into the polar contribution ∆G_PB/GB_ and the nonpolar contribution ∆G_nonpolar._

∆G_solvation_ = ∆G_PB/GB_ + ∆G_nonpolar_ (3)

The finite difference Poisson–Boltzmann (PB) or Generalized Born (GB) models were used to calculate the electrostatic solvation-free energy. The nonpolar component of the solvation-free energy was determined using the solvent-accessible surface area (SASA).

∆G_nonpolar_ = γ × ∆SASA + b (4)

To determine the solvent-accessible surface area, the linear combinations of pairwise overlaps (LCPO) approach was utilized. The solvation parameters γ and b for the PB approach are 0.00542 kcal/mol·Å^2^ and 0.92 kcal/mol, respectively, while for the GB method, they are 0.0072 kcal/mol·Å^2^ and 0 kcal/mol, respectively [50]. The solvent’s probe radius was set to 1.4 Å. PARSE radii were employed for the solvation model [51]. The quasi-harmonic entropy approximation was employed to calculate the conformational entropy change upon ligand binding (TΔS)*,* which includes the translational, rotational, and vibrational components. The single trajectory method was utilized to estimate the energy in this investigation. This method involves extracting the ligand and protein geometries from the protein–ligand complex, ensuring that the net molecular mechanical binding energy (ΔE_gas_) does not include any internal energy. Such energy estimation has been successfully applied in numerous investigations [52]. Due to sampling restrictions and significant fluctuations, the independent trajectory approaches, which involved three trajectories of the complex, free receptor, and free ligand, were found to be insufficient in practice for energy calculations.

## 3. Results

### 3.1. Microarray Data Analysis and DEG Identification

**Figure 2:**
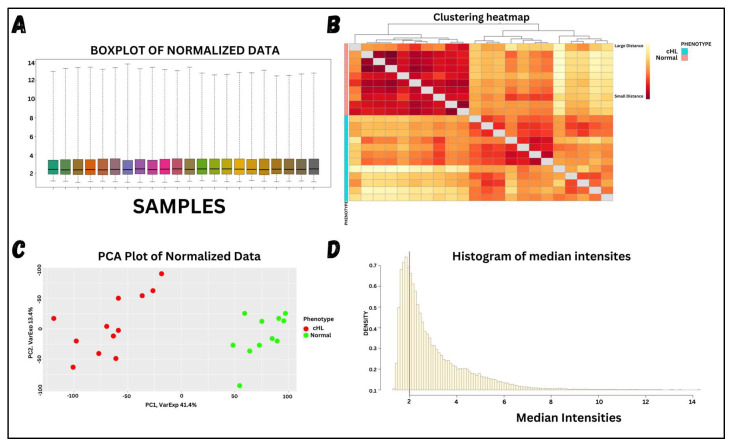
Visualizing processed data. (**A**) shows the boxplot of each sample, where all the samples have almost the same median, indicating the high quality of the data. This plot was generated using ggplot2 R-package v4.4.1. (**B**) shows a heatmap using Pheatmap R-package based on the normalized expression values of each sample, indicating correlations between them. Two clusters are visualized in the heatmap, showing that samples of the same phenotypes are closely correlated. (**C**) shows a PCA plot of the samples generated by ggplot2 R-package, where the classification of both phenotypes is observed, indicating significant expression differences. (**D**) shows a histogram of the median expression values across samples, with a threshold of 2 (red line) to ensure that only expression values greater than 2 are chosen.

**Figure 3:**
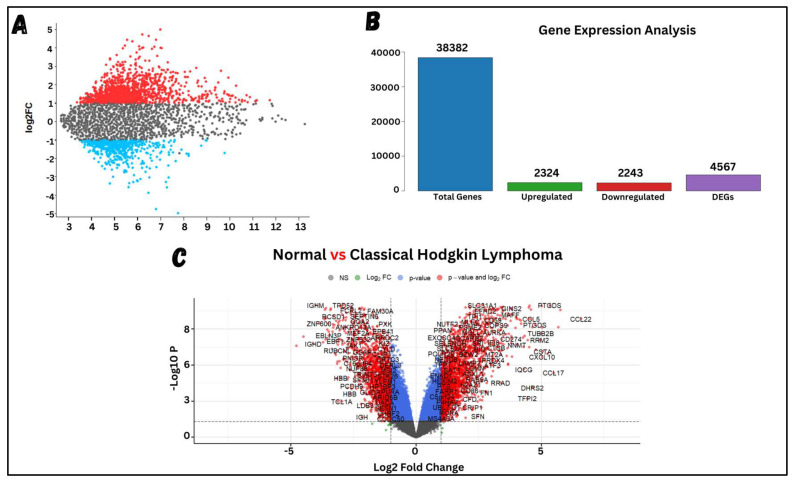
Visualization of significant DEGs. (**A**) shows an MA-plot where the average value of each gene is on the *X*-axis and logFC values on the *Y*-axis. The gray dots represent genes with logFC values below the threshold, i.e., (−1 ≤ logFC ≤ +1), while the red and blue dots represent genes with logFC values above the threshold, identified as DEGs. (**B**) shows the total number of genes and the final number of DEGs. (**C**) presents a volcano plot where non-significant genes are shown as gray dots, genes that are significant but do not meet the logFC threshold as blue dots, and significant genes that meet the logFC threshold as red dots.

Differential expression analysis of the microarray dataset generated a list of significant DEGs from the expression files. A statistical test was applied to clean the data, making it suitable for retrieving significant DEGs. Figure 2 shows the visualized form of the processed data. Examining the results, (A) shows the normalized data, where the median of all samples is equalized, making it suitable for DEG analysis. (B) displays the associations among samples based on the entire normalized expression values, highlighting a clear and significant difference between the cHL and healthy samples. (C) visualizes the expression values of each sample based on significant eigenvalues and vectors, showing clear and significant variation between the two phenotypes. These analyses were crucial to ensuring that the phenotypes have significantly different expression values, enabling the identification of appropriate DEGs. Lastly, (D) shows the chosen expression values for all samples to be analyzed in the final analysis. Expression values with median intensities below 2 were considered non-significant, as such low intensities often result from compromised scanner readings, potentially leading to the identification of non-significantly downregulated genes. After preprocessing the data and eliminating non-significant intensities, the clean data were subjected to DEG analysis, where significant DEGs were obtained based on the selection criteria of Log2FC (−1 ≥ logFC ≤ +1) and an adjusted *p*-value ≤ 0.05. The total number of genes, along with the DEGs, are visualized through various plots shown in Figure 3. A total of 38,382 genes were included in the study across all samples, of which 4567 genes were identified as DEGs—2324 upregulated and 2243 downregulated.

### 3.2. Pathway Enrichment Analysis of DEGs

**Figure 4:**
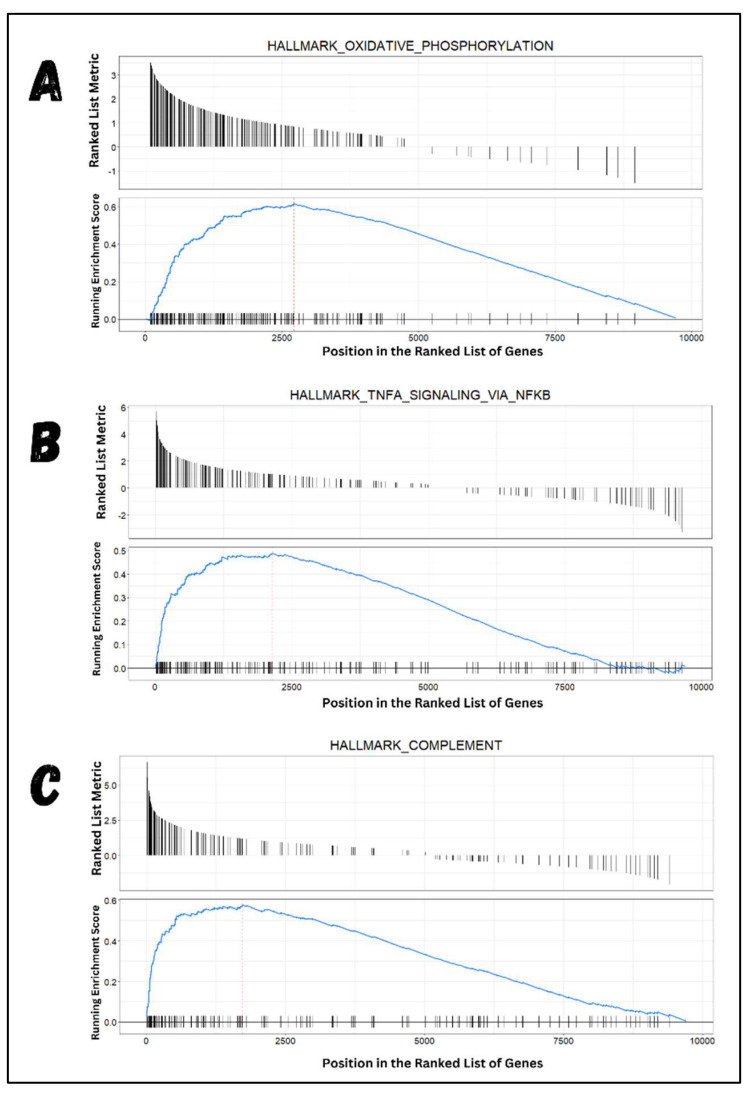
Top 3 GSEA pathways. Most of the top-regulated genes were associated with the mentioned pathways. As shown in (**A**–**C**), the ranked metric list denotes the ranked list provided as input, compared with the running enrichment score. Each image shows positive running enrichment scores, indicated by the running blue curve remaining high when it follows the upregulated genes across the ranked list and dropping gradually when downregulated genes were scanned on the row.

The obtained genes associated with logFC values were further used for pathway enrichment analysis to determine the involvement of DEGs in specific biological pathways. The total DEGs (4567), along with their specific logFC values, were arranged and used as an input file (ranked list) for the GSEA algorithm. The GSEA packages scanned the ranked gene list and associated it with known experimentally derived biological pathways. The results revealed that 37 pathways were positively enriched, as shown in Table 2. The top 3 pathways—hallmark oxidative phosphorylation, hallmark TNFA signaling via NFKB, and hallmark complement pathway—had the highest enrichment scores and included the most top-regulated genes, as shown in Figure 4. These gene sets associated with specific pathways were identified using a stringent statistical threshold of adjusted *p*-values of 0.02, underscoring the authenticity of the results. The analysis of the top 3 pathways indicates that they are highly regulated in experimentally derived cancer studies. These results suggest that many of the top-regulated genes in the ranked list are linked with biological pathways reported to be highly regulated in cancer cases.

### 3.3. Interactive Network Analysis

**Figure 5:**
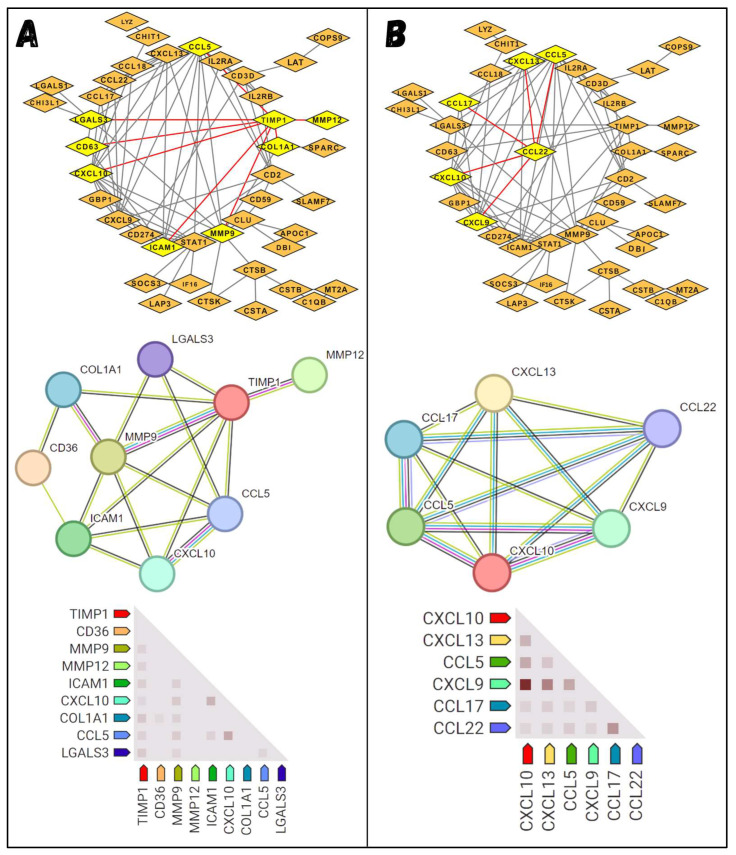
Interaction analysis. (**A**) shows the closest association of multiple genes with MMP12. MMP12 is highly associated with TIMP1 (the more interactive the colored lines, the stronger the interaction), which further interacts closely with other genes. The heatmap illustrates the coexpression and interdependence of different genes, where darker points in the heatmap indicate stronger coexpression relationships. (**B**) similarly depicts the close interactive genes associated with CCL22, along with the extent of their associations and coexpression.

Based on the previous analysis, interaction analysis was performed for the upregulated genes to examine their associations and relationships. Upregulated genes with logFC values above 2 were selected and used as input for network analysis. Upregulated genes with logFC values above 2 were selected and used as input for network analysis. Significant associations were visualized and narrowed down further by choosing the top 2 upregulated genes from the list. This step was chosen just to check the overall behavior and linkages of the top-upregulated genes. Narrowing down the analysis to the top most upregulated genes is significant because their pronounced behavior in cHL identifies them as key genes for further exploration. From the list of upregulated genes, matrix metallopeptidase 12 (MMP12) and C-C motif chemokine ligand 22 (CCL22), with the highest logFC values of 6.63782 and 6.583754, were chosen. Close associative genes with MMP12 and CCL22 were examined using the first neighbor function in Cytoscape. Figure 5A,B highlight the first neighbors of MMP12 and CCL22 in yellow. Additionally, all the close neighboring genes associated with MMP12 and CCL22 were further analyzed to determine the degree of interrelated associations. MMP12 shows a strong and significant link with TIMP1, which in turn shows a positive connection with MMP9 and other genes. Correlations specific to coexpression were visualized via a heatmap, providing a clearer depiction of the interrelationships among the genes. The same analysis was conducted for the CCL22 gene, revealing connections with several genes. The extent of these associations was further analyzed, showing strong associations with CCL17, CCL10, and CCL5, and the coexpressions were visualized by heatmap. Reviewing the literature revealed that the connecting members of MMP12 and CCL22 play significant roles in the progression of various malignancies, supporting the notion that MMP12 and CCL22 are targets of interest and can be used as potential therapeutic biomarkers in the case of cHL.

### 3.4. Evaluation of the Target Genes

**Figure 6:**
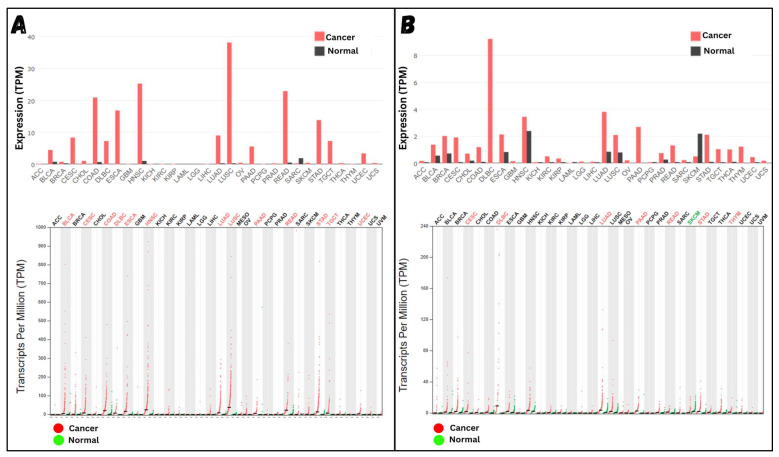
Reference Analysis: (**A**) shows the expression level of MMP12 in different malignancies. Out of a total of 31 malignancies, 13 show a reasonably high expression of MMP12. The accompanying dot plot also displays the expression levels, taking into account the reported number of individuals included in the case and control groups. (**B**) demonstrates similar results for CCL22, where high expression is reported in 17 malignancies.

**Figure 7:**
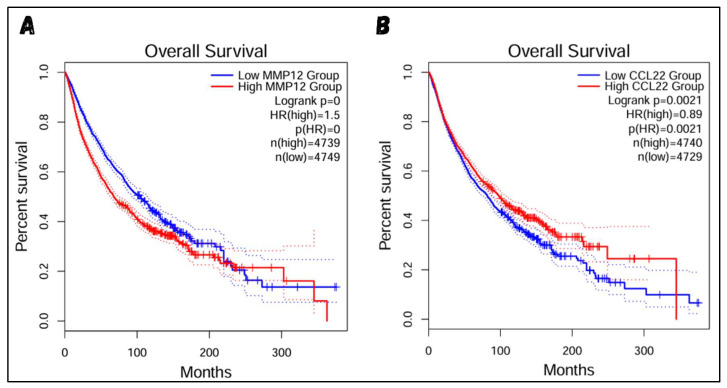
Survival Analysis: (**A**) shows the survival analysis of MMP12 and (**B**) CCL22. In both graphs, the blue slope represents the survival rate of individuals with low gene expression, while the red slope represents the survival rate of individuals with high gene expression. Survival analysis is performed on thousands of built-in reference datasets. If the majority of samples have a similar correlation with the query gene, they will be represented by bold lines (blue and red). Samples with slightly different correlations will be represented by dotted lines (blue and red).

The results strongly suggest that MMP12 and CCL22 should be considered therapeutic targets in cHL. To further support this hypothesis, both genes were cross-checked with existing expression data for MMP12 and CCL22 in various malignancies. Figure 6A,B show the expression levels of MMP12 and CCL22 across known data from normal and malignant individuals. The bar graphs and dot plots display the expression of both genes in transcripts per million (TPM), clearly illustrating the high expression of MMP12 and CCL22 in about 14 and 18 known malignancies, respectively. Considering the information from known experimental data, it is evident that MMP12 and CCL22, which show high expression in numerous malignancies, will also have high expression in cHL. To further validate this point, a survival analysis was conducted to evaluate the effect of active MMP12 and CCL22 expression on individual survival. The results in Figure 7A show that individuals with high MMP12 expression have a lower survival rate compared to those with low MMP12 expression. A drastic drop in survival is observed in the later months of the graph, clearly indicating the destructive nature of MMP12 when highly expressed. A similar trend is observed with CCL22 (Figure 7B). Although the survival rate of individuals with high CCL22 expression is greater than that of the lower expression group almost 75% of the time, a sudden and extreme drop in survival rate occurs after a specific duration, highlighting the sudden destructive nature of CCL22. From all the findings, it is evident that both genes are responsible for the progression of various malignancies and have harmful effects on individual health when highly expressed for a significant period of time. Although both genes have supportive evidence to be considered therapeutic targets, we will proceed with MMP12 due to its accurate structure availability and other important known properties. 

### 3.5. MMP12 Structure Analysis

**Figure 8:**
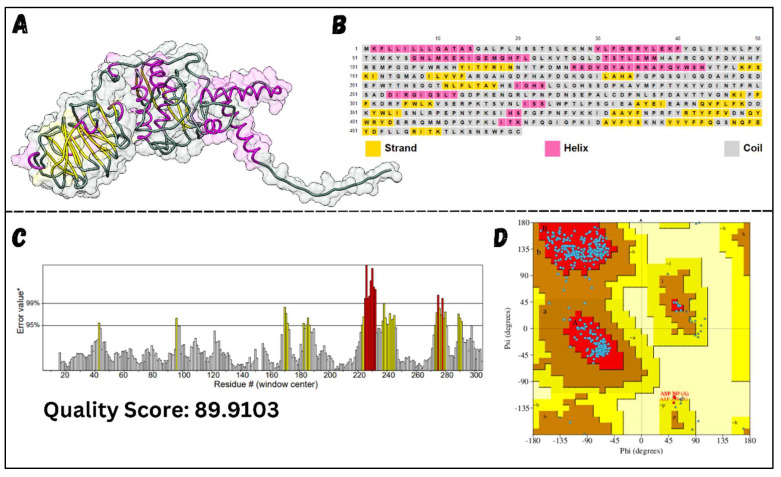
MMP12 Structure Assessment. (**A**) shows the three-dimensional structure of the protein, clearly highlighting helices, coils, and strands. (**B**) identifies the amino acids involved in forming helices, strands, and coils. (**C**) shows the ERRAT plot, demonstrating the stability of MMP12, with the majority of amino acids within the acceptable range. Although a few amino acids exceed the stability threshold (red), the overall ERRAT score remains acceptable. Yellow regions are those with 95% confidence of rejection (**D**) presents the Ramachandran plot, with most amino acids (blue dots) located in the most favored region (red), while two residues are in the disallowed region (pale yellow). Further details about the Ramachandran plot coloring and labelling can be found at https://doi.org/10.1002/pro.3289.

Upon retrieving the MMP12 structure from the UniProt database (MMP12 UniProt ID: P39900 · MMP12_HUMAN), it was refined, analyzed for secondary structure, and evaluated for stability using the Ramachandran and ERRAT algorithms, as shown in Figure 8. MMP12, formed from 470 residues, consists of a considerable number of helices and strands with fewer coils, making it one of the more stable protein families. The Ramachandran plot consists of four regions: most favored regions (red), additional allowed regions (brown), generously allowed regions (yellow), and disallowed regions (pale). The residues of the MMP12 construct were placed in these regions, and combined stability was calculated. The placement of residues in these regions is based on the Phi and Psi angles of each residue, which are plotted on the X-axis and Y-axis of the plot. Furthermore, ERRAT scores focus on the internal hydrogen bonds present between amino acid residues. Higher ERRAT scores indicate a greater number of hydrogen bonds within a protein, increasing its stability. Before structure refinement, 94.7% of the amino acids in MMP12 were in the most favored and additional allowed regions. To minimize steric clashes between residues, MMP12 was refined and re-evaluated, resulting in an increase to 99.2% of residues in the favored and allowed regions, as mentioned in Table 3. The ERRAT score was also considerably high, at 89.91, indicating MMP12 stability. This refinement step led to a relaxed structure, potentially optimizing docking.

### 3.6. Alpha-Based Active Site Prediction of MMP12 and Grid Coordinates Selection

**Figure 9:**
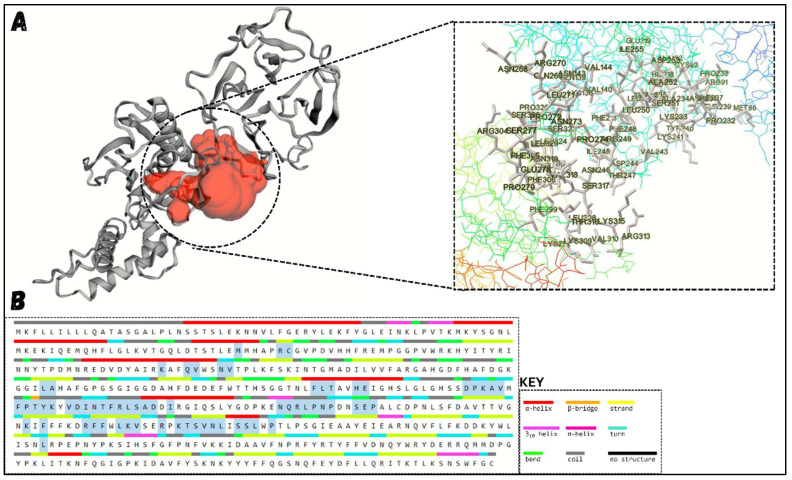
Predicted Active Site of MMP12. (**A**) shows the region of the active site in MMP12 (red) and its associated residues, while (**B**) represents the arranged residues involved in the active site (red) along with the overall secondary structure summary of the protein.

As MMP12 is an essential therapeutic target contributing to the progression of cHL, docking strategies were employed to inhibit its function by introducing anticancer compounds into its active site. Given that we are working with a modeled structure of MMP12, identifying the active site is necessary before ligand introduction. The active site and its associated amino acids were identified and are shown in Figure 9. This site involves 67 amino acids, as detailed in Supplementary Table S1, with a combined GRAVY score of −0.280, clearly indicating the hydrophilic nature of the active site.

**Figure 10:**
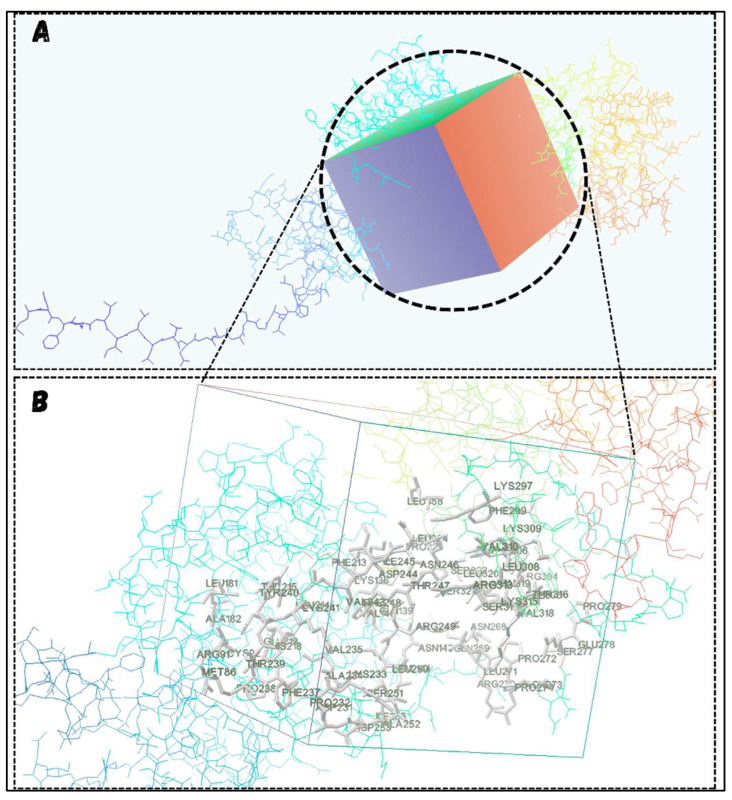
Setting the Grid Box. (**A**) shows the overall grid box covering the entire active site, while (**B**) visualizes the magnified transparent grid box and the involved residues of the active site.

Placing ligands at the exact active site of a protein is crucial for obtaining significant binding results. To achieve this, a grid box was set around the active sites, covering all the relevant residues, using the Autodock tool, as shown in Figure 10. The coordinates of the grid box covering the active sites are x = 4.939, y = −3.417, and z = 0.67, with the number of points in each dimension set to 62. This information guides the docking tool to dock the ligand specifically into the mentioned area.

### 3.7. Molecular Docking

**Figure 11:**
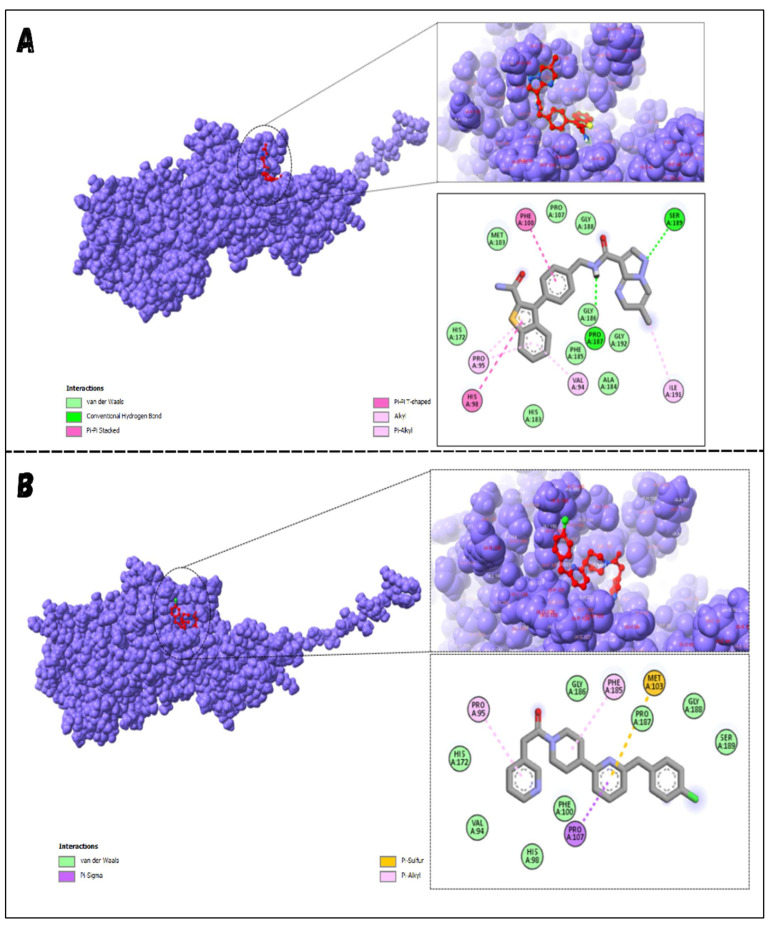
Docking Results. (**A**) shows the best binding pose of BDC_24037121 (red) into the active site of MMP12, with 16 interactions as indicated in the provided key. (**B**) shows the binding interactions of BDC_27854277 (red) with MMP12, totaling 12 interactions, making it the second-best ligand to be considered.

A specific library of targeted oncology-related compounds, retrieved from the Asinex database and consisting of over 4000 compounds, was docked into the active site of MMP12 using the PyRx docking tool [53]. The conformation limit for each compound was set to 50, meaning that each compound was docked into the active site with 50 acceptable poses. The selection of poses can vary according to the structure of the target protein [54]. A large library of docking conformations, along with their docking scores, was generated. The two ligands associated with the lowest ΔG were selected as the best ligands for interaction analysis. The two compounds, identified by their Asinex IDs BDC_24037121 and BDC_27854277, showed ΔG values of −7.7 kcal/mol and −7.6 kcal/mol, respectively, and were considered the top ligands with the highest binding affinity across the entire library. Docking results are shown in Figure 11, where ligand 1 (A) forms two hydrogen bonds and 13 non-hydrogen bonds with residues of the active site of MMP12. Ligand 2 (B) forms 12 non-hydrogen bonds with the active site residues of the target protein. Both ligands were evaluated for general and ADMET (adsorption, distribution, metabolism, excretion, and toxicity) properties, as mentioned in Table 4. Both ligands have values within acceptable ranges, making them suitable for bodily administration. 

### 3.8. Molecular Dynamic (MD) of Docked Complexes

**Figure 12:**
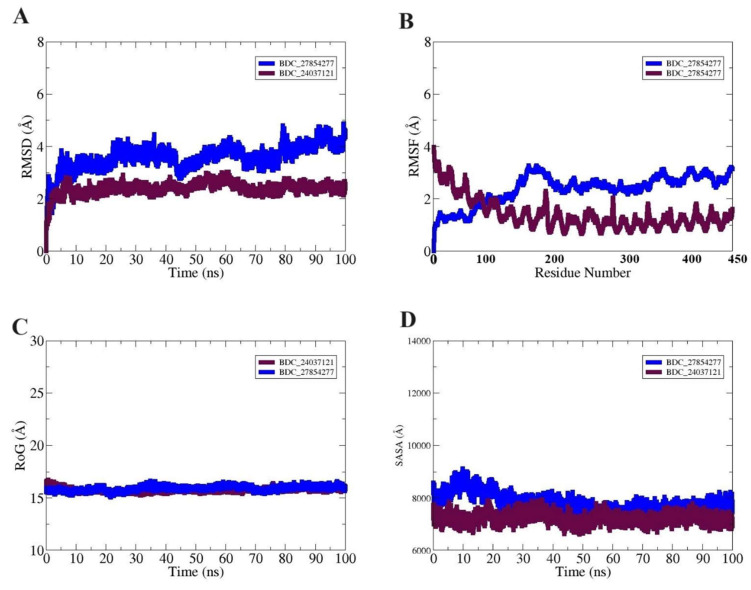
Simulations Results. (**A**) RMSD plot of MMP12 with BDC_24037121 and BDC_27854277 illustrates the stability of the complexes over time. (**B**) Residual fluctuations plot of MMP12 and the ligands after interactions show the dynamic behavior of the residues involved. (**C**) The linearly stable curve of the radius of gyration of docked complexes demonstrates the compactness and stability of the complexes. (**D**) SASA plot for the docked protein complex indicates changes in the solvent-accessible surface area, suggesting alterations in protein compaction due to ligand binding.

MD simulations aim to evaluate the binding behavior of ligands when docked to their receptor proteins. As mentioned above, all possible analyses were carried out for the docked complexes to assess their dynamic behavior, as shown in Figure 12. The root mean square deviations (RMSD), which measure deviations of all carbon alpha atoms using the docked complexes’ shape as a reference, were the first techniques used to assess structural stability. The results indicated that the docked complexes formed highly stable structures with only minor structural oscillations. In the active pocket, the inhibitor molecules remained stable, and both systems exhibited good convergence. The root mean square fluctuation (RMSF) of the complexes revealed significant fluctuations in the active site residues of MMP12, illustrating considerable interactions with the docked ligands. The radius of gyration (RoG) was employed to evaluate the overall residual fluctuation and compactness of the target protein when docked with a specific ligand. The uniformly stable curve of RoG observed in evaluating the docked complexes indicated the compactness and stability of the complexes. Additionally, analysis of the solvent-accessible surface area (SASA) showed a reduction, suggesting increased protein compaction due to drug binding.

### 3.9. Binding Free Energies Estimation

MMPBSA and MMGBSA were employed to validate the binding affinity of the docked ligands with MMP12. Both approaches were used simultaneously to validate and cross-check the binding affinities of the ligands with MMP12. Observing the affinities in Table 5, both complexes exhibited favorable energies associated with van der Waals and electrostatic interactions, indicating spontaneous and favorable binding of the ligands with MMP12. Additionally, the solvation energies of the complexes were relatively low in positive, suggesting that the introduction of solvent to the system displaces minimal energy from the vacuumed system. Lastly, the net binding energy showed large negative values, indicating that the ligands favorably bind to the active site of MMP12 and exhibit strong binding interactions.

## 4. Discussion

cHL is a type of lymphatic cancer that affects approximately 80,000 people worldwide each year [55]. With modern therapy, patients diagnosed at an early stage can expect five-year survival rates exceeding 90%. However, not everyone has access to such advanced care, and as a result, cHL causes over 25,000 deaths globally each year [55]. To address this issue, this study aimed to identify novel therapeutic targets by analyzing differential gene expression data from microarray gene expression profiles. Numerous studies have utilized this approach, leading to the identification of markers associated with various malignancies. In a study, detailed microarray data of different phenotypes like prostate cancer cell lines, prostate stromal cells, prostate cancer biopsies, and benign hyperplasia were studied, and highly expressed genes related to prostate cancer were identified that might be disease indicators or therapeutic targets [56]. Bittner et al. examined the expression of various genes in melanoma samples using a microarray dataset; wingless-related MMTV integration site 5A (WNT5A), which is associated with enhanced motility and invasiveness, emerged as one of the best predictive genes for identifying distinct sub-classes of melanoma [57,58,59].

The microarray gene expression dataset used in this study was solely related to cHL individuals, with a set of healthy individuals serving as a reference. None of the patients had been treated with chemotherapy or antibiotics, providing an unaltered representation of cHL patients. The data, retrieved from Affymetrix technology, was uploaded as unprocessed data. As shown in Supplementary Figure S1, the raw data were unrefined and contained irregularities, potentially resulting from dye bias, technical bias, ink bias, or array bias. These biases can lead to the inaccurate identification of DEGs. Normalization of microarray data is an obligatory step before identifying DEGs. For instance, a study performed RMA normalization on all microarray datasets related to pancreatic cancer and normal individuals before retrieving significant DEGs [60]. In the raw data of this study, the gene expression values had non-significant median values, and the cHL sample gene expressions were also not aligned. Additionally, the PCA plot showed poor classification of both phenotypes. RMA normalization was used in order to clean up the raw data and filter out false gene expression values. It is a statistical approach primarily used for microarray data and essential for deriving accurate gene expression values [18]. Raw data were processed, resulting in significant centered medians and clearly classified phenotypes (cHL and normal). The PCA plot showed highly distinct clusters for both phenotypes, indicating significant differences in gene expression values. PCA plots differentiate phenotypes based on variance across the gene expression values. The expression values of the mentioned phenotypes showed significant variance, clearly differentiating them into two significant clusters and illustrating distinctly different phenotypes. Additionally, box plots for each sample in the dataset displayed aligned medians, demonstrating the significant integrity and purity of the data. Furthermore, a correlation heatmap based on expression values revealed two major clusters corresponding to the phenotypes, clearly showing significant differences between the phenotypes.

The Limma package, designed for retrieving and identifying DEGs, was applied to the processed data. In total, 4567 DEGs were identified, consisting of 2243 downregulated and 2324 upregulated genes. The highest expression value (LogFC) among the top genes was 6.6378, while the lowest expression value was −6.5458. The top 8 upregulated genes identified were matrix metallopeptidase 12 (MMP12), C-C motif chemokine ligand 22 (CCL22), ectonucleotide pyrophosphatase/phosphodiesterase 2 (ENPP2), basic leucine zipper ATF-like transcription factor 3 (BATF3), members of the RAS oncogene family (RAB13), matrix metallopeptidase 9 (MMP9), and galectin 3 (LGALS3), having logFC values of 6.6378, 6.5837, 6.4091, 6.3778, 6.3397, 6.3131, and 5.9339, respectively. In a similar study proposed by [61], it was also concluded that MMP9 is highly expressed in cHL, having a logFC value of 6.1047, respectively. Other top-regulated genes having logFC values in the range of 5-3 were also reported, where the majority of the genes match our findings, e.g., metallopeptidase inhibitor (TIMP1) and C-C motif chemokine ligand-5 (CCL5).

GSEA analysis unveiled the active participation of upregulated genes in pathways linked to various malignancies. Among these from our list, 147 top-upregulated genes were found to be involved in the oxidative phosphorylation pathway, indicating a positive regulatory role in cHL. Previous studies have consistently highlighted the significant regulation of the oxidative phosphorylation pathway in different malignancies, suggesting its potential as a therapeutic target for cancer therapy [62,63,64]. Moreover, other established pathways, such as TNFA signaling via NFKB, complement pathway, and myc_targets_v1, among others, as listed in Supplementary Excel File S1, displayed significant regulation by upregulated genes from our dataset. A total of 121 highly expressed genes from our dataset were associated with TNFA signaling via the NFKB pathway. TNFA signaling via the NFKB pathway, for instance, has been noted for its high expression in Hodgkin lymphoma, with associated genes implicated in various cancer-related processes such as cell proliferation, JAK/STAT pathway activation, loss of B-cell marker expression, cellular interaction, and a positive NFkB feedback loop [65]. Additionally, 102 upregulated genes from our list were linked to the upregulation of the complement pathway, which has been identified as an active pathway involved in cancer progression [66]. 

Network analysis revealed an interesting association between the upregulated genes. A total of 42 genes with logFC values greater than or equal to 3 were evaluated for interactions, resulting in the identification of the most significant interactions among the top-regulated genes. Among the resulting interactions, MMP12 and CCL22 were also present, which were the top two upregulated genes on the list. The significant top-positive regulation of these genes in cHL makes them focal points for further analysis. ing genes associated with MMP12 and CCL22 were explored, where MMP12 showed direct interaction with MMP9, TIMP1, collagen type I alpha 1 chain (COL1A1), LGALS3, CXCL10, intercellular adhesion molecule 1 (ICAM1), and C-C motif chemokine ligand 5 (CCL5), while CCL22 showed the closest association with C-X-C motif chemokine 13 (CXCL13), CXCL9, CXCL10, CCL5, and CCL17, respectively. All the associated genes associated with MMP12 and CCL22 had logFC values in the range of 6.6–3.2. 

MMP12 is a protein that has been reported to be highly expressed in various malignancies and plays a significant role in cancer progression [67,68,69,70]. The associated genes with MMP12 were further reviewed, revealing significant associations with other genes that play active roles in cancer progression. A study related to gastric cancer indicated that MMP12 and COL1A1 collectively promote cancer progression. Additionally, the protein-protein interaction network analysis in this study revealed an interaction between COL1A1 and MMP12, suggesting a potential functional connection between these two genes in the context of cancer progression [71]. LGALS3 plays a significant role in cancer progression by influencing immune responses. The interaction between LGALS3 and MMP12 is notable, as LGALS3 is cleaved and activated by MMPs. This interaction halts the apoptosis of cancer cells, promoting cancer cell survival and contributing to cancer progression [72]. The collective role of MMP9 and MMP12, along with other MMPs, has been reported as effective therapeutic and prognostic biomarkers in various malignancies. These MMPs contribute to tumor invasion, metastasis, and angiogenesis, making them valuable targets for cancer therapy and important indicators for cancer prognosis [73,74]. TIMP1 is known to inhibit MMPs and plays an active role in cancer repression [67]. However, in our case, the expression value of TIMP1 is half the expression value of MMPs, strongly suggesting that its expression may be suppressed by the upregulation of MMPs. This imbalance between MMPs and TIMP1 could contribute to the enhanced cancer progression observed in our study. CCL5 (derived from glioma-associated microglia/brain macrophages [GAMs]) enhances glioma cell invasiveness through a novel calcium-dependent MMP2 signaling pathway [75]. CXCL10 has been reported to have significant roles in various cancers when produced in high amounts. In our case, its expression exceeds a logFC value of 5, which might indicate a progressive role in cHL [76]. MMP12 showed a significant connection with CXCL10, suggesting a cooperative and combined role in cHL progression. MMP12 is a strong cancer progressor, and its significant association with other cancer progressor genes makes it a key gene to be considered a therapeutic target against cHL. CCL22, the second most highly regulated gene, has been reported to play significant roles in various cancer progressions. It has been used as a therapeutic target in different experimental studies, where its blockage through various strategies has resulted in cancer repression [77,78,79,80]. Its associated genes in the network present in our data showed supportive roles in the progression of different cancers, making it a key gene to be considered therapeutic target [81].

MMP12, considered an essential target, was explored further for structural analysis because docking and simulation strategies were intended for this research. The goal was to block MMP12 with known experimental anticancer compounds. This approach aims to investigate the inhibitory functions of these compounds, which can then be further validated in experimental studies. Multiple studies have demonstrated strategies where the target gene of interest is examined, refined, validated, and then docked with compounds of interest. This process aims to identify potential inhibitors that can effectively bind to the target gene and disrupt its function, offering a pathway for developing new therapeutic agents [82,83,84]. Docking studies were performed using the anticancer compounds library, where a significant binding score was witnessed with respect to two compounds having significant interactions with active site residues of MMP12 and a binding RMSD score of 3.613 and 3.798, making it the best fit for MMP12. Simulation studies also supported the docking results, showing supportive bindings and stable docked complexes, respectively. 

Our study underscores the pivotal role of MMP12 as a cancer progressor in various malignancies, including cHL, making it a focal point for investigation and a potential therapeutic target. Through comprehensive analyses, we identified significant interactions and explored known therapeutic agents as potential inhibitors against MMP12. However, our study has certain limitations, including the lack of exploration into downregulated genes, which represents a notable gap that warrants further investigation. Additionally, given the proven therapeutic significance of CCL22 in cHL and other malignancies, it should be considered for docking and simulation studies in future research endeavors. The findings of our study provide a foundation for further validation through additional deep analysis and experimental validation of docking and simulation results. 

## 5. Conclusions

cHL is a significant malignancy, with numerous cases annually in both Western and Asian communities. Despite effective chemotherapy, relapse remains a problem, indicating a need for more specific treatments. Identifying therapeutic targets associated with cHL progression is crucial. Analyzing gene expression data helps identify regulated genes for functional and pathway analysis, revealing specific targets that could impact the disease. Our study focused on MMP12 as a key player in cHL progression. We proposed inhibiting MMP12 by docking known anticancer compounds into its active site, offering a specific therapeutic strategy for cHL patients. This biomarker and inhibitory compound need experimental validation through in vitro and in vivo studies to ensure their efficacy and safety for human use. Validating the proposed biomarker and inhibitory compound through experimental studies is essential to developing more specific and effective treatments for cHL patients.

## Figures and Tables

**Table 1:** Sample information related to the GSE12453 dataset.

Accession No	Total Samples	Selected Samples	Platform	Country
GSE12453	67 samples	22 samples 10 Healthy 12 cHL	Affymetrix Human Genome U133 Plus 2.0 Array	Germany
Detailed Information of Samples				
Source Name		Phenotype		Expression Files
cHL1		Classical Hodgkin lymphoma		GSM312811
cHL2		Classical Hodgkin lymphoma		GSM312812
cHL3		Classical Hodgkin lymphoma		GSM312813
cHL4		Classical Hodgkin lymphoma		GSM312814
cHL5		Classical Hodgkin lymphoma		GSM312815
cHL6		Classical Hodgkin lymphoma		GSM312816
cHL7		Classical Hodgkin lymphoma		GSM312817
cHL8		Classical Hodgkin lymphoma		GSM312818
cHL9		Classical Hodgkin lymphoma		GSM312819
cHL10		Classical Hodgkin lymphoma		GSM312820
cHL11		Classical Hodgkin lymphoma		GSM312821
cHL12		Classical Hodgkin lymphoma		GSM312822
Normal1		Normal		GSM312870
Normal2		Normal		GSM312872
Normal3		Normal		GSM312874
Normal4		Normal		GSM312875
Normal5		Normal		GSM312876
Normal6		Normal		GSM312877
Normal7		Normal		GSM312879
Normal8		Normal		GSM312882
Normal9		Normal		GSM312883
Normal10		Normal		GSM312886

**Table 2:** GSEA-derived pathways. The positively enriched pathways were derived from the ranked list. A total of 37 pathways are positively enriched where their description, net enrichment score (NES), and number of genes (set size) involved in the pathway are shown.

S.No	Description	NES	Set Size
1	HALLMARK_OXIDATIVE_PHOSPHORYLATION	3.259831	147
2	HALLMARK_TNFA_SIGNALING_VIA_NFKB	2.916283	121
3	HALLMARK_COMPLEMENT	2.866847	102
4	HALLMARK_EPITHELIAL_MESENCHYMAL TRANSITION	2.829082	82
5	HALLMARK_COAGULATION	2.732839	56
6	HALLMARK_MTORC1_SIGNALING	2.722621	128
7	HALLMARK_E2F_TARGETS	2.708754	143
8	HALLMARK_INFLAMMATORY_RESPONSE	2.669728	102
9	HALLMARK_ADIPOGENESIS	2.650821	115
10	HALLMARK_MYC_TARGETS_V1	2.594028	140
11	HALLMARK_IL6_JAK_STAT3_SIGNALING	2.585827	52
12	HALLMARK_GLYCOLYSIS	2.572058	102
13	HALLMARK_ALLOGRAFT_REJECTION	2.568368	112
14	HALLMARK_APOPTOSIS	2.551558	102
15	HALLMARK_IL2_STAT5_SIGNALING	2.540642	119
16	HALLMARK_INTERFERON_GAMMA_RESPONSE	2.527682	128
17	HALLMARK_UV_RESPONSE_UP	2.491597	81
18	HALLMARK_DNA_REPAIR	2.290871	95
19	HALLMARK_XENOBIOTIC_METABOLISM	2.282905	91
20	HALLMARK_MYC_TARGETS_V2	2.273425	40
21	HALLMARK_HYPOXIA	2.262949	107
22	HALLMARK_P53_PATHWAY	2.251974	116
23	HALLMARK_REACTIVE_OXYGEN_SPECIES PATHWAY	2.230879	31
24	HALLMARK_CHOLESTEROL_HOMEOSTASIS	2.202292	41
25	HALLMARK_ESTROGEN_RESPONSE_LATE	2.182724	95
26	HALLMARK_FATTY_ACID_METABOLISM	2.100737	78
27	HALLMARK_KRAS_SIGNALING_UP	2.056103	85
28	HALLMARK_G2M_CHECKPOINT	1.962562	137
29	HALLMARK_UNFOLDED_PROTEIN_RESPONSE	1.942093	74
30	HALLMARK_INTERFERON_ALPHA_RESPONSE	1.841332	58
31	HALLMARK_APICAL_JUNCTION	1.764329	85
32	HALLMARK_BILE_ACID_METABOLISM	1.688062	43
33	HALLMARK_MYOGENESIS	1.676596	90
34	HALLMARK_PROTEIN_SECRETION	1.644965	53
35	HALLMARK_PEROXISOME	1.580537	55
36	HALLMARK_HEME_METABOLISM	1.520337	104
37	HALLMARK_ANDROGEN_RESPONSE	1.517247	59

**Table 3:** Statistics of Ramachandran Plot. This table illustrates the maximum number of residues in the most favored regions of the plot that represent the maximum stability of MMP12.

Parameter/Region	Refined Ppk1 Protein	
	No. of Residues	Percentage
Most favored regions (A, B, C)	389	95.8%
Additional allowed regions (a, b, l, p)	14	3.4%
Generously allowed regions (~a, ~b, ~1, ~p)	1	0.2%
Disallowed regions (XX)	2	0.5%
Non-glycine and non-proline residues	406	100%
End-residues (excl. Gly and pro)	2	
Glycine residues	35	
Proline residues	27	
Total Number of residues	470	

**Table 4:** Ligands structure and chemical properties. Both the ligands, along with their Asinex IDs, chemical properties, structures, and web presentation of additional properties, are mentioned.

Asinex ID	BDC_24037121	BDC_27854277	Structure
Formula	C24H24ClN3O	C24H31N5O2S	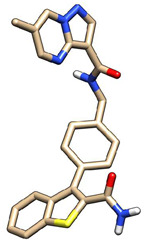
Molecular Weight	405.92	453.6	
Hydrogen Bond Donor	0	2	
Hydrogen Bond Acceptor	3	4	
Heavy atoms	29	32	
Lipophilicity	4.11	1.68	
BBB-permeability	Yes	No	
GI-absorption	High	High	
Pgp substrate	Yes	Yes	
CYP1A2 inhibitor	Yes	No	
CYP2C19 inhibitor	Yes	No	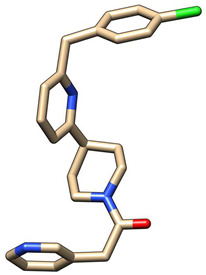
CYP2C9 inhibitor	Yes	No	
CYP2D6 inhibitor	Yes	No	
CYP3A4 inhibitor	Yes	Yes	
Lipinski violations	No violations	No violations	
Ghose violations	No violations	No violations	
Veber violations	No violations	No violations	
Bioavailability Score	0.55	0.55	
PAINS alerts	0 alerts	1 alert	
hERG Blocker	No	No	
Lead likeness violations	2	1	
Synthetic accessibility	Moderate (2.96)	High (4.92)	
Visualized accepted properties			
BDC_24037121		BDC_27854277	
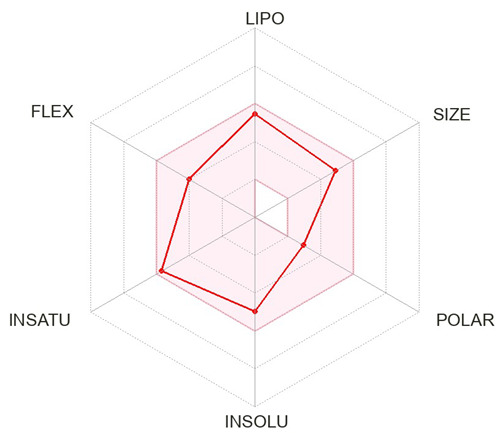		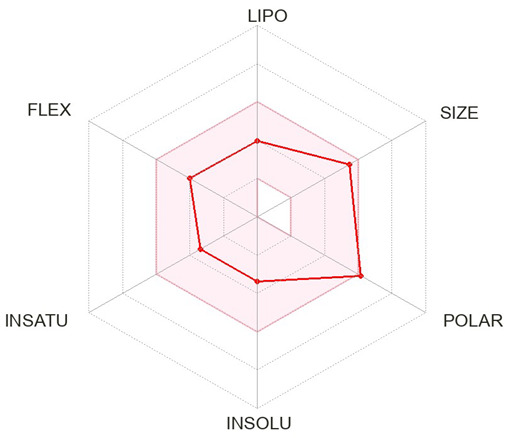	

**Table 5:** Validating binding affinities. MMGBSA and MMPBSA binding energies are estimated for each docked complex.

Parameter	MMP12-BDC_24037121	MMP12-BDC_27854277
MMGBSA		
VDWAALS	−69.34	−45.87
Electrostatic Interactions	−24.09	−14.28
DELTA G solvation	15.35	11.36
DELTA TOTAL	−78.08	−48.79
MMPBSA		
VDWAALS	−69.34	−45.87
Electrostatic Interactions	−24.09	−14.28
DELTA G solvation	11.38	10.48
DELTA TOTAL	−82.05	−49.67

## Data Availability

All the data generated in the current research is presented in the manuscript.

## Supplementary Materials

The following supporting information can be downloaded at: https://www.mdpi.com/article/10.3390/molecules29153476/s1. Figure S1: Visualization of RAW data. Table S1: Active site residues along with its position and coordinates. Excel File S1.

## References

[1] Achkova D. (2016). Development of Immunotherapy for Classical Hodgkin’s Lymphoma and Anaplastic Large Cell Lymphoma Using Colony-Stimulating Factor-1 Receptor Re-Targeted T-Lymphocytes.

[2] Huang X., Nolte I., Gao Z., Vos H., Hepkema B., Poppema S., van den Berg A., Diepstra A. (2011). Epidemiology of Classical Hodgkin Lymphoma and Its Association with Epstein Barr Virus in Northern China. PLoS ONE.

[3] Liu D. (2017). Hodgkin Lymphoma. Tumors and Cancers.

[4] Gao J., Chen Y., Wu P., Wang F., Tao H., Shen Q., Wang S., Gong S., Zhang X., Zhou Z. (2021). Causes of Death and Effect of Non-Cancer-Specific Death on Rates of Overall Survival in Adult Classic Hodgkin Lymphoma: A Populated-Based Competing Risk Analysis. BMC Cancer.

[5] Gupta S., Craig J.W. (2023). Classic Hodgkin Lymphoma in Young People. Semin. Diagn. Pathol..

[6] Mohty R., Dulery R., Bazarbachi A.H., Savani M., Hamed R.A., Bazarbachi A., Mohty M. (2021). Latest Advances in the Management of Classical Hodgkin Lymphoma: The Era of Novel Therapies. Blood Cancer J..

[7] Beg A., Parveen R. (2021). Role of Bioinformatics in Cancer Research and Drug Development. Translational Bioinformatics in Healthcare and Medicine.

[8] Desany B., Zhang Z. (2004). Bioinformatics and Cancer Target Discovery. Drug Discov. Today.

[9] Brenner C. (2019). Applications of Bioinformatics in Cancer. Cancers.

[10] Wang W., He Y., Zhao Q., Zhao X., Li Z. (2020). Identification of Potential Key Genes in Gastric Cancer Using Bioinformatics Analysis. Biomed. Rep..

[11] Nagl S. (2006). Cancer Bioinformatics: From Therapy Design to Treatment.

[12] Wu D., Rice C.M., Wang X. (2012). Cancer Bioinformatics: A New Approach to Systems Clinical Medicine. BMC Bioinform..

[13] Shi J., Walker M.G. (2007). Gene Set Enrichment Analysis (GSEA) for Interpreting Gene Expression Profiles. Curr. Bioinform..

[14] Szklarczyk D., Kirsch R., Koutrouli M., Nastou K., Mehryary F., Hachilif R., Gable A.L., Fang T., Doncheva N.T., Pyysalo S. (2023). The STRING Database in 2023: Protein–Protein Association Networks and Functional Enrichment Analyses for Any Sequenced Genome of Interest. Nucleic Acids Res..

[15] Shannon P., Markiel A., Ozier O., Baliga N.S., Wang J.T., Ramage D., Amin N., Schwikowski B., Ideker T. (2003). Cytoscape: A Software Environment for Integrated Models of Biomolecular Interaction Networks. Genome Res..

[16] Weinstein J.N., Collisson E.A., Mills G.B., Shaw K.R., Ozenberger B.A., Ellrott K., Shmulevich I., Sander C., Stuart J.M. (2013). The Cancer Genome Atlas Pan-Cancer Analysis Project. Nat. Genet..

[17] Lonsdale J., Thomas J., Salvatore M., Phillips R., Lo E., Shad S., Hasz R., Walters G., Garcia F., Young N. (2013). The Genotype-Tissue Expression (GTEx) Project. Nat. Genet..

[18] Kim C.S., Hwang S., Zhang S.-D. Rma with Quantile Normalization Mixes Biological Signals between Different Sample Groups in Microarray Data Analysis. Proceedings of the 2014 IEEE International Conference on Bioinformatics and Biomedicine (BIBM).

[19] Brune V., Tiacci E., Pfeil I., Döring C., Eckerle S., Van Noesel C.J.M., Klapper W., Falini B., von Heydebreck A., Metzler D. (2008). Origin and Pathogenesis of Nodular Lymphocyte—Predominant Hodgkin Lymphoma as Revealed by Global Gene Expression Analysis. J. Exp. Med..

[20] Liu S., Wang Z., Zhu R., Wang F., Cheng Y., Liu Y. (2021). Three Differential Expression Analysis Methods for RNA Sequencing: Limma, EdgeR, DESeq2. JoVE (J. Vis. Exp.).

[21] Johnson W.E., Li C., Rabinovic A. (2007). Adjusting Batch Effects in Microarray Expression Data Using Empirical Bayes Methods. Biostatistics.

[22] Tsai C.-A., Chen Y.-J., Chen J.J. (2003). Testing for Differentially Expressed Genes with Microarray Data. Nucleic Acids Res..

[23] Witten D., Tibshirani R. (2007). A Comparison of Fold-Change and the t-Statistic for Microarray Data Analysis. Analysis.

[24] Chen J.J., Wang S.J., Tsai C.A., Lin C.J. (2007). Selection of Differentially Expressed Genes in Microarray Data Analysis. Pharmacogenomics J..

[25] Yu G., Wang L.-G., Han Y., He Q.-Y. (2012). ClusterProfiler: An R Package for Comparing Biological Themes among Gene Clusters. Omics A J. Integr. Biol..

[26] Dolgalev I. (2020). Msigdbr: Msigdb Gene Sets for Multiple Organisms in a Tidy Data Format. R Package Version 7.2.1. https://igordot.github.io/msigdbr/authors.html.

[27] Suarez-Farinas M., Lowes M.A., Zaba L.C., Krueger J.G. (2010). Evaluation of the Psoriasis Transcriptome across Different Studies by Gene Set Enrichment Analysis (GSEA). PLoS ONE.

[28] Wickham H., Chang W., Wickham M.H. (2016). Package ‘Ggplot2’. Creat. Elegant Data Vis. Using Gramm. Graph. Version.

[29] Tang Z., Li C., Kang B., Gao G., Li C., Zhang Z. (2017). GEPIA: A Web Server for Cancer and Normal Gene Expression Profiling and Interactive Analyses. Nucleic Acids Res..

[30] Shen Y., Liu J., Zhang L., Dong S., Zhang J., Liu Y., Zhou H., Dong W. (2019). Identification of Potential Biomarkers and Survival Analysis for Head and Neck Squamous Cell Carcinoma Using Bioinformatics Strategy: A Study Based on TCGA and GEO Datasets. BioMed Res. Int..

[31] Consortium U. (2019). UniProt: A Worldwide Hub of Protein Knowledge. Nucleic Acids Res..

[32] Lee G.R., Won J., Heo L., Seok C. (2019). GalaxyRefine2: Simultaneous Refinement of Inaccurate Local Regions and Overall Protein Structure. Nucleic Acids Res..

[33] Laskowski R.A. (2022). PDBsum 1: A Standalone Program for Generating PDBsum Analyses. Protein Sci..

[34] Colovos C., Yeates T.O. (1993). Verification of Protein Structures: Patterns of Nonbonded Atomic Interactions. Protein Sci..

[35] Dundas J., Ouyang Z., Tseng J., Binkowski A., Turpaz Y., Liang J. (2006). CASTp: Computed Atlas of Surface Topography of Proteins with Structural and Topographical Mapping of Functionally Annotated Residues. Nucleic Acids Res..

[36] Dhameliya T.M., Nagar P.R., Gajjar N.D. (2022). Systematic Virtual Screening in Search of SARS-CoV-2 Inhibitors against Spike Glycoprotein: Pharmacophore Screening, Molecular Docking, ADMET Analysis and MD Simulations. Mol. Divers..

[37] Pettersen E.F., Goddard T.D., Huang C.C., Couch G.S., Greenblatt D.M., Meng E.C., Ferrin T.E. (2004). UCSF Chimera—A Visualization System for Exploratory Research and Analysis. J. Comput. Chem..

[38] Ganguly A., Tsai H.-C., Fernández-Pendás M., Lee T.-S., Giese T.J., York D.M. (2022). AMBER Drug Discovery Boost Tools: Automated Workflow for Production Free-Energy Simulation Setup and Analysis (ProFESSA). J. Chem. Inf. Model..

[39] Huey R., Morris G.M., Forli S. (2012). Using AutoDock 4 and AutoDock Vina with AutoDockTools: A Tutorial. Scripps Res. Inst. Mol. Graph. Lab..

[40] Ounthaisong U., Tangyuenyongwatana P. (2017). Cross-Docking Study of Flavonoids against Tyrosinase Enzymes Using PyRx 0.8 Virtual Screening Tool. TJPS.

[41] Jejurikar B.L., Rohane S.H. (2021). Drug Designing in Discovery Studio. https://www.proquest.com/docview/2532716945?sourcetype=Scholarly%20Journals.

[42] Case D.A., Aktulga H.M., Belfon K., Ben-Shalom I., Brozell S.R., Cerutti D.S., Cheatham T.E., Cruzeiro V.W.D., Darden T.A., Duke R.E. (2021). Amber 2021.

[43] Bayly C.I., Cieplak P., Cornell W., Kollman P.A. (1993). A Well-Behaved Electrostatic Potential Based Method Using Charge Restraints for Deriving Atomic Charges: The RESP Model. J. Phys. Chem..

[44] Wang J., Wolf R.M., Caldwell J.W., Kollman P.A., Case D.A. (2004). Development and Testing of a General Amber Force Field. J. Comput. Chem..

[45] Jorgensen W.L., Chandrasekhar J., Madura J.D., Impey R.W., Klein M.L. (1983). Comparison of Simple Potential Functions for Simulating Liquid Water. J. Chem. Phys..

[46] Debiec K.T., Cerutti D.S., Baker L.R., Gronenborn A.M., Case D.A., Chong L.T. (2016). Further along the Road Less Traveled: AMBER Ff15ipq, an Original Protein Force Field Built on a Self-Consistent Physical Model. J. Chem. Theory Comput..

[47] Darden T., York D., Pedersen L. (1993). Particle Mesh Ewald: An N⋅ Log (N) Method for Ewald Sums in Large Systems. J. Chem. Phys..

[48] Laskowski R.A., Swindells M.B. (2011). LigPlot+: Multiple Ligand—Protein Interaction Diagrams for Drug Discovery. J. Chem. Inf. Model..

[49] Wang J., Morin P., Wang W., Kollman P.A. (2001). Use of MM-PBSA in Reproducing the Binding Free Energies to HIV-1 RT of TIBO Derivatives and Predicting the Binding Mode to HIV-1 RT of Efavirenz by Docking and MM-PBSA. J. Am. Chem. Soc..

[50] Weiser J., Shenkin P.S., Still W.C. (1999). Approximate Atomic Surfaces from Linear Combinations of Pairwise Overlaps (LCPO). J. Comput. Chem..

[51] Sitkoff D., Sharp K.A., Honig B. (1994). Accurate Calculation of Hydration Free Energies Using Macroscopic Solvent Models. J. Phys. Chem..

[52] Cong X.-J., Tan J.-J., Liu M., Chen W.-Z., Wang C.-X. (2010). Computational Study of Binding Mode for N-Substituted Pyrrole Derivatives to HIV-1 Gp41. Prog. Biochem. Biophys..

[53] Adebayo G.P., Oduselu G.O., Aderohunmu D.V., Klika K.D., Olasehinde G.I., Ajani O.O., Adebiyi E. (2024). Structure-Based Design, and Development of Amidinyl, Amidoximyl and Hydroxamic Acid Based Organic Molecules as Novel Antimalarial Drug Candidates. Arab. J. Chem..

[54] Mishra S., Kumar S., Ramdas, Khare S., Shukla A., Shanker K., Pal A., Khan F., Darokar M.P. (2023). Quebrachitol from Putranjiva Roxburghii Wall.(Putranjivaceae) a Potent Antimalarial: Pre-Clinical Efficacy and Its Interaction with PfLDH. Parasitol. Int..

[55] Hjalgrim H., Jarrett R.F. (2020). Epidemiology of Hodgkin Lymphoma. Hodgkin Lymphoma. Hematologic Malignancies.

[56] Welsh J.B., Sapinoso L.M., Su A.I., Kern S.G., Wang-Rodriguez J., Moskaluk C.A., Frierson H.F., Hampton G.M. (2001). Analysis of Gene Expression Identifies Candidate Markers and Pharmacological Targets in Prostate Cancer. Cancer Res..

[57] Bittner M., Meltzer P., Chen Y., Jiang Y., Seftor E., Hendrix M., Radmacher M., Simon R., Yakhini Z., Ben-Dor A. (2000). Molecular Classification of Cutaneous Malignant Melanoma by Gene Expression Profiling. Nature.

[58] Carr K.M., Bittner M., Trent J.M. (2003). Gene-Expression Profiling in Human Cutaneous Melanoma. Oncogene.

[59] Weeraratna A.T., Jiang Y., Hostetter G., Rosenblatt K., Duray P., Bittner M., Trent J.M. (2002). Wnt5a Signaling Directly Affects Cell Motility and Invasion of Metastatic Melanoma. Cancer Cell.

[60] Nisar M., Paracha R.Z., Arshad I., Adil S., Zeb S., Hanif R., Rafiq M., Hussain Z. (2021). Integrated Analysis of Microarray and RNA-Seq Data for the Identification of Hub Genes and Networks Involved in the Pancreatic Cancer. Front. Genet..

[61] Kuang Z., Guo L., Li X. (2017). Identification of Key Genes and Pathways Associated with Classical Hodgkin Lymphoma by Bioinformatics Analysis. Mol. Med. Rep..

[62] Sica V., Bravo-San Pedro J.M., Stoll G., Kroemer G. (2020). Oxidative Phosphorylation as a Potential Therapeutic Target for Cancer Therapy. Int. J. Cancer.

[63] Ashton T.M., McKenna W.G., Kunz-Schughart L.A., Higgins G.S. (2018). Oxidative Phosphorylation as an Emerging Target in Cancer Therapy. Clin. Cancer Res..

[64] Nayak A.P., Kapur A., Barroilhet L., Patankar M.S. (2018). Oxidative Phosphorylation: A Target for Novel Therapeutic Strategies against Ovarian Cancer. Cancers.

[65] Jardin F. (2022). NFkB Pathway and Hodgkin Lymphoma. Biomedicines.

[66] Kleczko E.K., Kwak J.W., Schenk E.L., Nemenoff R.A. (2019). Targeting the Complement Pathway as a Therapeutic Strategy in Lung Cancer. Front. Immunol..

[67] Salmela M.T., Karjalainen-Lindsberg M.-L., Puolakkainen P., Saarialho-Kere U. (2001). Upregulation and Differential Expression of Matrilysin (MMP-7) and Metalloelastase (MMP-12) and Their Inhibitors TIMP-1 and TIMP-3 in Barrett’s Oesophageal Adenocarcinoma. Br. J. Cancer.

[68] Hofmann H.-S., Hansen G., Richter G., Taege C., Simm A., Silber R.-E., Burdach S. (2005). Matrix Metalloproteinase-12 Expression Correlates with Local Recurrence and Metastatic Disease in Non–Small Cell Lung Cancer Patients. Clin. Cancer Res..

[69] Li G.-S., Tang Y.-X., Zhang W., Li J.-D., Huang H.-Q., Liu J., Fu Z.-W., He R.-Q., Kong J.-L., Zhou H.-F. (2024). MMP12 Is a Potential Predictive and Prognostic Biomarker of Various Cancers Including Lung Adenocarcinoma. Cancer Control.

[70] Lv F.Z., Wang J.L., Wu Y., Chen H.F., Shen X.Y. (2015). Knockdown of MMP12 Inhibits the Growth and Invasion of Lung Adenocarcinoma Cells. Int. J. Immunopathol. Pharmacol..

[71] Liu S., Chen L., Zeng J., Chen Y. (2023). A Prognostic Model Based on the COL1A1-Network in Gastric Cancer. Am. J. Transl. Res..

[72] Farhad M., Rolig A.S., Redmond W.L. (2018). The Role of Galectin-3 in Modulating Tumor Growth and Immunosuppression within the Tumor Microenvironment. Oncoimmunology.

[73] Li K., Li D., Hafez B., Bekhit M.M.S., Jardan Y.A.B., Alanazi F.K., Taha E.I., Auda S.H., Ramzan F., Jamil M. (2024). Identifying and Validating MMP Family Members (MMP2, MMP9, MMP12, and MMP16) as Therapeutic Targets and Biomarkers in Kidney Renal Clear Cell Carcinoma (KIRC). Oncol. Res..

[74] Impola U., Uitto V.-J., Hietanen J., Hakkinen L., Zhang L., Larjava H., Isaka K., Saarialho-Kere U. (2004). Differential Expression of Matrilysin-1 (MMP-7), 92 KD Gelatinase (MMP-9), and Metalloelastase (MMP-12) in Oral Verrucous and Squamous Cell Cancer. J. Pathol. A J. Pathol. Soc. Great Br. Irel..

[75] Wu C.Y.-J., Chen C.-H., Lin C.-Y., Feng L.-Y., Lin Y.-C., Wei K.-C., Huang C.-Y., Fang J.-Y., Chen P.-Y. (2018). CCL5 from Tumor-Associated Macrophages/Microglia (TAMs) Regulates Glioma Migration and Invasion via Calcium-Dependent Matrix Metalloproteinase-2. Cancer Res..

[76] Liu M., Guo S., Stiles J.K. (2011). The Emerging Role of CXCL10 in Cancer. Oncol. Lett..

[77] Anz D., Rapp M., Eiber S., Koelzer V.H., Thaler R., Haubner S., Knott M., Nagel S., Golic M., Wiedemann G.M. (2015). Suppression of Intratumoral CCL22 by Type i Interferon Inhibits Migration of Regulatory T Cells and Blocks Cancer Progression. Cancer Res..

[78] Sun J., Sun J., Song B., Zhang L., Shao Q., Liu Y., Yuan D., Zhang Y., Qu X. (2016). Fucoidan Inhibits CCL22 Production through NF-κB Pathway in M2 Macrophages: A Potential Therapeutic Strategy for Cancer. Sci. Rep..

[79] Lecoq I., Kopp K.L., Chapellier M., Mantas P., Martinenaite E., Perez-Penco M., Rønn Olsen L., Zocca M.-B., Wakatsuki Pedersen A., Andersen M.H. (2022). CCL22-Based Peptide Vaccines Induce Anti-Cancer Immunity by Modulating Tumor Microenvironment. Oncoimmunology.

[80] Mukaida N., Sasaki S., Baba T. (2014). Chemokines in Cancer Development and Progression and Their Potential as Targeting Molecules for Cancer Treatment. Mediat. Inflamm..

[81] Do H.T.T., Lee C.H., Cho J. (2020). Chemokines and Their Receptors: Multifaceted Roles in Cancer Progression and Potential Value as Cancer Prognostic Markers. Cancers.

[82] Aloufi B.H. (2022). Structure-Based Multi-Targeted Molecular Docking and Molecular Dynamic Simulation Analysis to Identify Potential Inhibitors against Ovarian Cancer. J. Biochem. Technol..

[83] Jha V., Devkar S., Gharat K., Kasbe S., Matharoo D.K., Pendse S., Bhosale A., Bhargava A. (2022). Screening of Phytochemicals as Potential Inhibitors of Breast Cancer Using Structure Based Multitargeted Molecular Docking Analysis. Phytomedicine Plus.

[84] Acharya R., Chacko S., Bose P., Lapenna A., Pattanayak S.P. (2019). Structure Based Multitargeted Molecular Docking Analysis of Selected Furanocoumarins against Breast Cancer. Sci. Rep..

