# Supplementary material for: Insights into the Gene Expression Profile of Classical Hodgkin Lymphoma: A Study towards Discovery of Novel Therapeutic Targets
Source: Molecules. 2024 Jul 25;29(15):3476. doi: 10.3390/molecules29153476 (PMC11314437; doi:10.3390/molecules29153476)
Supplement: Supplementary file 1 [file molecules-29-03476-s001.zip › Supplementary Tables and Figures.pdf]

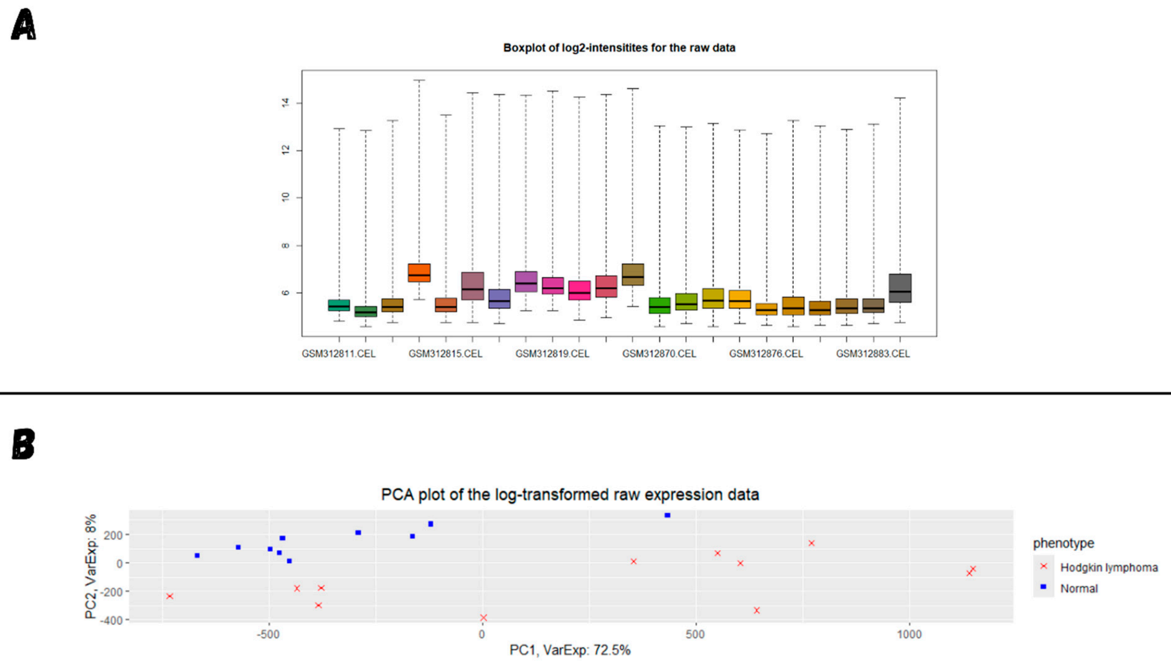

Supplementary figure S1: Visualization of RAW data.

Supplementary Table S1: Active site residues along with its position and coordinates.

Amino Acids	Residue No#	X-axis	Y-axis	Z-axis
MET	86	14.96	-8.106	-16.684
ARG	91	11.36	-0.22	-15.638
CYS	92	13.248	2.746	-12.331
LYS	136	-0.853	-1.647	1.829
GLN	139	3.029	2.857	8.824
VAL	140	6.107	0.291	2.211
ASN	143	6.309	1.955	9.397
VAL	144	12.089	-1.468	7.457
LEU	181	6.71	0.41	-14.392
ALA	182	8.256	6.293	-12.073
PHE	213	3.556	-0.693	-0.371
LEU	214	5.936	0.443	-6.523
THR	215	6.692	3.449	-10.397
HIS	218	13.638	0.489	-8.829
GLU	219	10.774	4.682	-10.309
ASP	231	17.884	-9.836	-2.859
PRO	232	13.186	-9.484	-7.67

LYS	233	15.172	-15.436	-2.898
ALA	234	13.345	-5.314	-5.85
VAL	235	9.989	-1.863	-4.179
PHE	237	15.221	-7.054	-12.185
PRO	238	13.083	-1.142	-13.121
THR	239	11.797	-5.597	-15.361
TYR	240	4.996	-1.974	-10.859
LYS	241	8.703	-12.675	-10.534
VAL	243	5.277	-6.842	-4.887
ASP	244	1.768	-8.145	-1.878
ILE	245	0.721	-5.18	0.434
ASN	246	0.283	-8.523	2.886
THR	247	2.47	-10.876	-0.868
PHE	248	7.339	-3.942	-2.155
ARG	249	6.787	-9.302	7.252
LEU	250	11.878	-1.305	3.217
SER	251	14.739	-7.541	-0.456
ALA	252	17.674	-9.133	3.615
ASP	253	18.178	-6.821	1.236
ILE	255	14.062	-5.235	5.763
ASN	268	8.119	2.222	18.461
GLN	269	7.383	4.734	13.544
ARG	270	9.756	-3.081	17.321
LEU	271	7.931	-5.031	12.31
PRO	272	8.559	-6.239	17.141
ASN	273	12.134	-10.609	17.899
PRO	274	13.085	-11.169	14.431
SER	277	6.45	-10.652	20.779
GLU	278	8.213	-15.212	15.914
PRO	279	3.731	-12.632	20.085
LYS	297	-4.793	-11.922	0.606
PHE	299	-5.46	-9.972	6.594
ARG	304	4.773	-2.77	20.66
PHE	305	4.75	-7.786	17.202
PHE	306	-0.95	-8.158	9.098
LEU	308	-1.876	-12.682	5.276
LYS	309	-3.151	-20.881	8.96
VAL	310	-2.984	-16.855	3.543
ARG	313	1.763	-17.716	-1.932
LYS	315	9.398	-19.28	6.475
THR	316	3.843	-17.609	9.665

SER	317	2.234	-13.565	7.389
VAL	318	3.718	-14.417	13.909
ASN	319	3.892	-9.58	8.573
LEU	320	4.139	-4.729	15.768
SER	322	2.667	-0.91	13.314
SER	323	4.283	-4.191	8.109
LEU	324	-2.802	-6.47	6.445
PRO	326	0.132	2.287	7.484
LEU	358	-5.635	-8.011	2.404
